# Therapeutic Potential of Exportin 1 and Aurora Kinase A Inhibition in Multiple Myeloma Cells

**DOI:** 10.3390/hematolrep18010010

**Published:** 2026-01-09

**Authors:** Seiichi Okabe, Yuko Tanaka, Shunsuke Otsuki, Mitsuru Moriyama, Seiichiro Yoshizawa, Akihiko Gotoh, Daigo Akahane

**Affiliations:** Department of Hematology, Tokyo Medical University, Tokyo 1600023, Japan; yukois9@yahoo.co.jp (Y.T.); mitsuru.c.moriyama@gmail.com (M.M.); b_perfect_simple@yahoo.co.jp (S.Y.); akgotou@juntendo.ac.jp (A.G.); daigoakahane@msn.com (D.A.)

**Keywords:** multiple myeloma, XPO1 inhibitor, aurora kinase inhibitor, plasma cell leukemia

## Abstract

**Background/Objectives:** Aurora kinases (AURKs) are key regulators of mitosis, and their dysregulation contributes to plasma cell disorders, including multiple myeloma (MM) and plasma cell leukemia (PCL). **Methods:** The expression and prognostic relevance of AURK family members were examined, and the therapeutic potential of AURKA inhibition was evaluated. **Results:** Gene expression analysis demonstrated significant upregulation of AURKA in PCL. Treatment of MM cells with the selective AURKA inhibitor LY3295668 induced dose-dependent cytotoxicity, caspase-3/7 activation, and cellular senescence. Similarly, selinexor, a selective exportin-1 inhibitor, elicited dose-dependent cytotoxicity and apoptosis. Combined treatment with LY3295668 and selinexor significantly improved apoptosis compared with either agent alone, and AURKA knockdown further sensitized MM cells to selinexor, thereby increasing apoptosis. In bortezomib-resistant MM cells and primary PCL samples, the combination therapy induced cytotoxicity and caspase-3/7 activation. **Conclusions:** These findings underscore AURKA expression as a prognostic marker in plasma cell disorders and support the therapeutic potential of combining AURKA inhibition with selinexor for bortezomib-resistant MM and PCL. To explore biomarker-driven strategies for optimizing therapeutic outcomes, future studies are warranted.

## 1. Introduction

Multiple myeloma (MM) is a common hematologic malignancy characterized by clonal plasma cell expansion and excessive production of monoclonal immunoglobulins, with increasing incidence worldwide [1]. MM typically develops from monoclonal gammopathy of undetermined significance (MGUS), an asymptomatic precursor condition [2]. Although their causal relationship remains unclear, progression from MGUS to MM has been associated with accumulating genetic alterations and changes in the bone marrow microenvironment [3]. Plasma cell leukemia (PCL), a rare and aggressive variant of plasma cell dyscrasia, accounts for 2–4% of MM cases and is linked to poor prognosis and distinct cytogenetic abnormalities [4,5].

MM diagnosis requires a comprehensive evaluation, including laboratory testing, imaging, and bone marrow biopsy. According to the International Myeloma Working Group, symptomatic MM is defined by the presence of ≥10% clonal plasma cells in the bone marrow with at least one myeloma-defining event, such as hypercalcemia, renal impairment, anemia, or lytic bone lesions (CRAB features) or specific biomarkers predictive of organ damage [1]. Early detection of these markers is essential to facilitate timely intervention and enhance clinical outcomes. Advances in proteasome inhibitors, immunomodulatory drugs, and monoclonal antibodies have markedly prolonged survival in patients with MM [6]. Natural killer cells are key innate immune effectors that mediate cytotoxicity against malignant cells. In MM, decreased natural killer cell activity has been associated with advanced disease, adverse clinical features, and inferior survival [7]. Furthermore, tumor necrosis factor-α (TNF-α) plays a dual role in MM, promoting B-cell proliferation while also inducing myeloma cell death. Elevated serum TNF-α is associated with increased disease activity [8].

Selinexor, an oral selective inhibitor of nuclear export, targets exportin 1 (XPO1), a key mediator of cytoplasmic transport for tumor suppressor proteins, such as p53 and Forkhead box O. XPO1 overexpression has been observed in MM, making it a promising therapeutic target [9]. Inhibition of XPO1 by selinexor disrupts oncogenic signaling, induces apoptosis, and has been approved for the treatment of relapsed or refractory MM. Nonetheless, therapeutic resistance often develops, limiting long-term efficacy and highlighting the need for rational drug combination strategies to improve clinical benefit [9]. In patients receiving oral selinexor, plasma exposure is characterized by a T_max_ of approximately 2–4 h, a mean C_max_ ranging from 0.5 to 0.7 μg/mL (1–1.5 μM) at doses of 60–80 mg, and an apparent terminal half-life of approximately 6–8 h [10].

Aurora kinases (AURKs) are serine/threonine kinases that play a central role in mitotic progression, spindle assembly, and chromosome alignment [11,12]. The AURK family includes AURKA, AURKB, and AURKC, with AURKA and AURKB frequently overexpressed across a wide range of malignancies, including MM [13]. Particularly, AURKA functions as a synthetic lethal partner of several tumor suppressors and plays a critical role in the G2/M transition, mitotic spindle formation, and DNA replication [14]. These critical functions have made AURKs attractive therapeutic targets, leading to the development of several specific inhibitors [15]. In a previous study, one AURKA inhibitor, LY3295668, achieved plasma concentrations of 6000 ng/mL (11 µM) at clinically relevant doses, in line with pharmacokinetic data from early-phase clinical trials [16].

Because selinexor is clinically available for relapsed/refractory MM and as AURKA is overexpressed in malignancies, we hypothesized that dual inhibition of nuclear export and mitotic regulation could represent a rational therapeutic strategy for these aggressive disease subsets. Thus, our study aimed to evaluate the antimyeloma activity of selinexor in combination with the AURKA inhibitor LY3295668 and to explore the therapeutic relevance of AURKA inhibition in MM and PCL, especially in high-risk and treatment-refractory settings.

## 2. Materials and Methods

### 2.1. Reagents

Selinexor (KPT-330, ATG-010) and AURKA inhibitor LY3295668 (AK-01), a selective AURKA inhibitor, were obtained from Selleck Chemicals (Houston, TX, USA). Stock solutions were prepared in dimethyl sulfoxide. All other reagents were purchased from Merck KGaA (Darmstadt, Germany).

### 2.2. Cell Lines, Cell Culture, and Primary Samples

U266 and RPMI8226 cells were obtained from ATCC (Manassas, VA, USA), and the bortezomib-resistant KMS-11/BTZ cell line was purchased from the JCRB Cell Bank (Osaka, Japan). The establishment and characterization of the KMS-11/BTZ cell line have been previously described [17]. Cells were cultured in RPMI 1640 medium supplemented with 10% fetal bovine serum and 1% penicillin–streptomycin and maintained at 37 °C in a humidified incubator under an atmosphere of 5% CO_2_. Peripheral blood samples were collected from a single patient at the time of PCL diagnosis after written informed consent, with approval from the Institutional Review Board of Tokyo Medical University (T2023-0105). Peripheral blood mononuclear cells were isolated by density-gradient centrifugation using Lymphocyte Separation Medium 1077 (PromoCell, Heidelberg, Germany) and immediately processed for analysis. Circulating plasma cells comprised 12% of total plasma cells during diagnosis. To evaluate the temporal changes in the measured parameters, additional peripheral blood samples were collected at three predefined time points during the same clinical course. Serial blood sampling at this frequency is part of routine clinical practice in our department and was conducted concurrently with clinically indicated blood tests.

### 2.3. Data Collection and Processing

Microarray data from Gene Expression Omnibus (GEO) dataset GSE13591, including gene expression profiles of plasma cells from normal donors (*n* = 5) and patients with MGUS (*n* = 11), MM (*n* = 133), or PCL (*n* = 9) [18], were analyzed using GEO2R. Differentially expressed genes were identified according to a log_2_(fold-change) of ≥1.0 and a *p*-value of <0.05. False discovery rate (FDR) was adjusted using the Benjamini–Hochberg procedure. AURKA mRNA expression profiles in myeloma and other hematological malignancies were retrieved from the Dependency Map (DepMap) portal (https://depmap.org/portal accessed on 10 October 2025) and compared using normalized RNA-seq expression values.

### 2.4. Cell Viability and Apoptosis Assays

A total of 2 × 10^5^ MM cells were treated for 72 h with selinexor alone, LY3295668 alone, or two different combinations of selinexor and LY3295668. Cell viability was evaluated using the trypan blue exclusion method and Cell Counting Kit-8 (Dojindo Laboratories, Kumamoto, Japan). Absorbance was measured using a Revvity Nivo™ multimode microplate reader (Revvity, Waltham, MA, USA). Drug interactions (synergy, additivity, or antagonism) were evaluated using the Chou-Talalay method, and combination index (CI) values were calculated from the dose-response curves [19]. Apoptotic cells were quantified through Annexin V staining followed by BD FACSLyric™ flow cytometer (BD Biosciences, San Jose, CA, USA) or BD Accuri™ C6 flow cytometer (BD Biosciences, San Jose, CA, USA) [20]. At least 10,000 events were acquired for apoptosis analysis. To ensure reproducibility, each experiment was independently performed at least three times.

### 2.5. Cytotoxicity and Senescence Assays

A total of 2 × 10^5^ MM cells/mL were treated with the indicated concentrations of selinexor or LY3295668 for 48 h or 72 h. Cytotoxicity was evaluated by measuring lactate dehydrogenase (LDH) release using the Cytotoxicity LDH Assay Kit (Dojindo). The importance of LDH determination in clinical and experimental oncology, underscoring its roles as a biomarker of tumor burden, cell damage/necrosis, and treatment response [21]. Cellular senescence was assessed through β-galactosidase staining (Cell Signaling Technology, Danvers, MA, USA), and stained cells were counted under a microscope (Olympus Corporation, Tokyo, Japan). Each experiment was independently performed at least three times to ensure reproducibility.

### 2.6. Quantitative Reverse Transcription Polymerase Chain Reaction and RNA Interference

Quantitative reverse transcription polymerase chain reaction (RT-qPCR) was conducted using SYBR Green reagents (Roche Diagnostics GmbH, Mannheim, Germany) on a LightCycler 2.0 system, with AURKA and β-actin primers (Takara Bio Inc., Kusatsu, Shiga, Japan).

In this study, the sequences of all primers used are listed below:

AURKA forward: 5′-CAGGCAACCAGTGTACCTCATC-3′

AURKA reverse: 5′-GAGGGCGACCAATTTCAAAG-3′

β-actin forward: 5′-TGGCACCCAGCACAATGAA-3′

β-actin reverse: 5′-CTAAGTCATAGTCCGCCTAGAAGCA-3′

AURKA knockdown was attained using small hairpin RNA (shRNA) lentiviral vectors (VectorBuilder, Guangzhou, China). U266 cells were infected with these vectors, and AURKA expression levels were quantified by RT-qPCR and immunoblotting. The target sequence is demonstrated in bold, with flanking sequences in regular font.

5′-AAGTTTATTCTGGCTCTTAAAGTGTTATTTAAAGCTCAGCT-3′, 5′-ATTTCCTTGTCAGAATCCATTACCTGTAAATAGTGGCCAGG-3′. The sequence of the scrambled shRNA used as a negative control was 5′-CCTAAGGTTAAGTCGCCCTCG-3′. Each experiment was independently conducted at least three times to ensure reproducibility.

### 2.7. Immunoblotting

Immunoblotting was conducted as previously described [22]. After treatment, cells were harvested via centrifugation and lysed by sonication in radioimmunoprecipitation assay buffer. Protein concentrations were measured using a Bio-Rad Protein Assay Kit (Bio-Rad Laboratories, Hercules, CA, USA). Equal amounts of lysate (40-µg total protein) were separated on 4–20% polyacrylamide gels and subsequently transferred to polyvinylidene difluoride membranes. Membranes were probed with primary antibodies against PLK1 (sc-17783, Santa Cruz Biotechnology, Dallas, TX, USA), phospho-γH2AX (05-636, Millipore, Burlington, MA, USA), β-actin (Santa Cruz Biotechnology), cleaved caspase-3 (#9661, Cell Signaling Technology), and Aurora A (#4718, Cell Signaling Technology). Primary antibodies were incubated at the manufacturer-recommended dilutions for 2 h at room temperature. Protein bands were visualized using enhanced chemiluminescence with the Amersham ECL kit (GE Healthcare, Tokyo, Japan). Immunoblot bands were quantified through densitometric analysis using ImageJ software (version 1.54; National Institutes of Health, Bethesda, MD, USA).

### 2.8. Cell Cycle Analysis

Cell cycle analysis was conducted using the BD CycleTest™ Plus DNA Reagent Kit (Becton Dickinson, Mountain View, CA, USA) according to the manufacturer’s instructions. Using the 100 nM selinexor and/or 100 nM LY3295668, 2 × 10^5^ cells/mL of U266 cells were cultured for 24 h. At least 10,000 events were acquired for each sample. Cell cycle distribution was analyzed using a BD FACSLyric™ flow cytometer (BD Biosciences, San Jose, CA, USA) or BD Accuri™ C6 flow cytometer (BD Biosciences), and data were processed using FlowJo™ software (version 7.6; FlowJo LLC, Ashland, OR, USA).

### 2.9. Statistical Analysis

All statistical analyses were performed using GraphPad Prism 10 (version 10.0.1; GraphPad Software, San Diego, CA, USA) using two-tailed tests. Comparisons between two groups were performed using Student’s *t*-test; when variances were unequal, Welch’s *t*-test was employed. For non-normally distributed data, the Mann-Whitney U test was used. Comparisons among ≥3 groups were conducted using one- or two-way analysis of variance, followed by Tukey’s honestly significant difference test for all pairwise comparisons or the Holm-Šidák for prespecified contrasts. Data were expressed as the mean ± standard deviation. Exact *p*-values were reported where available. Statistical significance was defined as *p*-values of <0.05 (*), <0.01 (**), <0.001 (***), and <0.0001 (****).

## 3. Results

### 3.1. Expression and Prognostic Significance of AURKs in Plasma Cell Disorders

**Figure 1:**
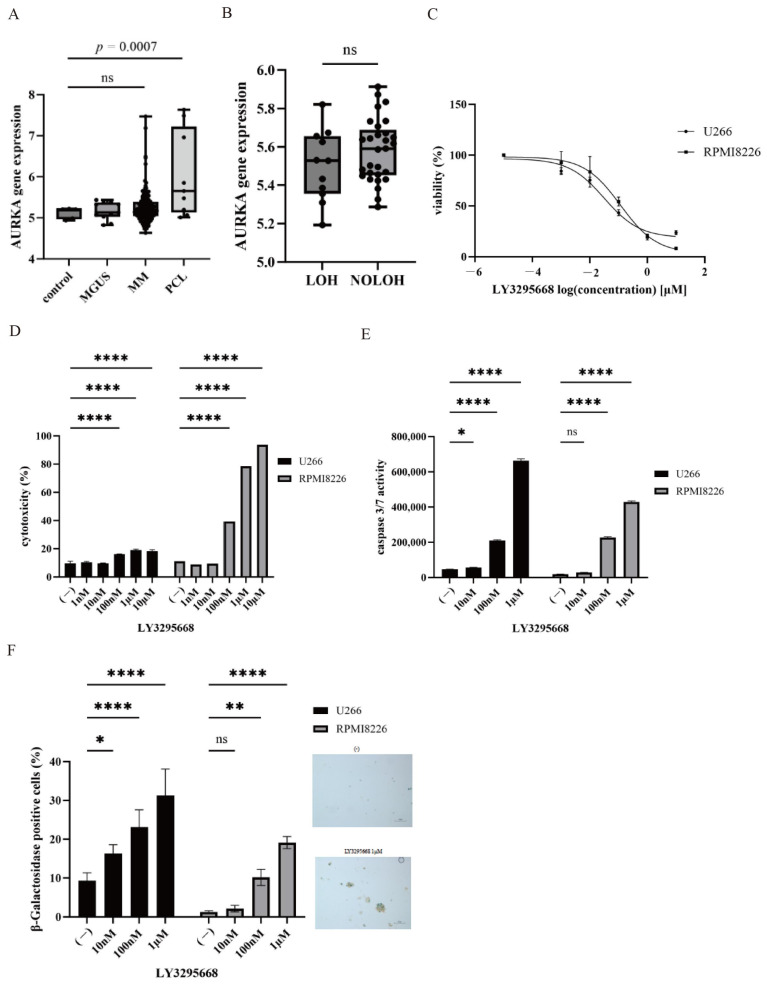
Expression and prognostic significance of AURKs in plasma cell disorders (**A**) *AURKA* expression levels were analyzed using data from the GEO database, GSE13591. A significant increase was noted compared with plasma cells from normal donors. (**B**) *AURKA* expression stratified by LOH status (LOH vs. nLOH) using GSE13591 data showed no significant difference (ns). (**C**) Multiple myeloma cell lines (U266 and RPMI-8226) were cultured with LY3295668 (0 nM–10 µM) for 72 h. Cell viability was evaluated using the Cell Counting Kit-8 assay (*n* = 3). (**D**) Multiple myeloma cell lines (U266 and RPMI-8226) were cultured with LY3295668 (0–10 µM) for 48 h. Cytotoxicity was assessed using a Cytotoxicity LDH Assay Kit. Data were normalized to untreated controls and presented as the mean  ±  standard deviation. **** *p*  < 0.0001, compared with the control (*n* = 3). (**E**) Multiple myeloma cell lines (U266 and RPMI-8226) cells were treated with LY3295668 (0–1 µM) for 48 h. Caspase-3/7 activity measured to evaluate apoptosis. * *p*  < 0.05 and **** *p*  < 0.0001, compared with the control sample (ns: not significant) (*n* = 3). (**F**) Multiple myeloma cell lines (U266 and RPMI-8226) were cultured with LY3295668 (0 nM–1 µM) for 72 h. SA-β-gal activity was assessed using the SA-β-gal Staining Kit (Cell Signaling Technology, Inc., Danvers, MA, USA) according to the manufacturer’s instructions. Representative micrographs of multiple myeloma cells, either untreated or treated with LY3295668 (1 µM). The scale bar represents 10 μm. * *p*  < 0.05, ** *p*  < 0.01, **** *p*  < 0.0001, compared with the control sample (ns: not significant) (*n* = 3).

The gene expression profiles of AURK family members were analyzed using the GEO database to examine their relevance in plasma cell disorders. A significant upregulation of AURKA and AURKB was noted in primary PCL samples compared with plasma cells from normal donors, whereas AURKC expression was significantly reduced in PCL (Figure 1A). Analysis of loss of heterozygosity (LOH) status (GSE13591) demonstrated no significant association with AURKA expression (Figure 1B). Survival analyses could not be performed because the limited clinical information available in GSE13591. We also compared AURKA expression in myeloma with that in other hematological malignancies using publicly available transcriptomic datasets and found no significant difference (Figure S1a). To further evaluate the therapeutic potential of AURKA inhibition, MM cell lines were treated with the AURKA inhibitor LY3295668. LY3295668 treatment induced dose-dependent cytotoxicity in MM cells (Figure 1C,D). The estimated IC50 concentrations of LY3295668 were approximately 0.3 µM for U266 cells and 0.18 µM for RPMI8226 cells. Furthermore, caspase-3/7 activity was increased following treatment, indicating activation of apoptotic pathways (Figure 1E). We utilized different dose ranges of LY3295668 because its dynamic response window differed between cytotoxicity and caspase 3/7 assays. We also noted enhanced senescence-associated β-galactosidase (SA-β-gal) staining, indicating the induction of cellular senescence (Figure 1F). Collectively, these findings show the efficacy of LY3295668 in targeting AURKA in MM and its potential to trigger apoptosis and senescence in malignant plasma cells.

### 3.2. Efficacy of Selinexor in Myeloma Cell Lines

**Figure 2:**
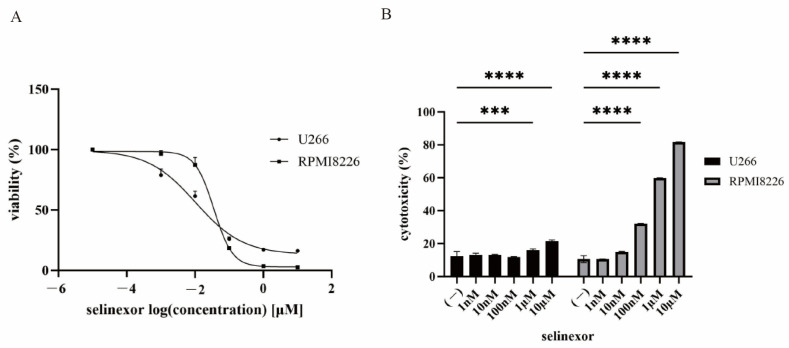
Effects of selinexor on MM cell lines (**A**) MM cell lines (U266 and RPMI8226) were cultured in RPMI 1640 medium containing the indicated concentration of selinexor for 72 h. Cell viability was assessed using the Cell Counting Kit-8 (*n* = 3). (**B**) MM cell lines (U266 and RPMI8226) were treated with the indicated concentration of selinexor for 48 h. Cytotoxicity was subsequently assessed utilizing the Cytotoxicity LDH Assay Kit. *** *p*  < 0.001 and **** *p*  < 0.0001, compared with the control sample (*n* = 3).

We next examined the efficacy of selinexor against MM cells. Treatment with selinexor resulted in a dose-dependent reduction in cell viability (Figure 2A). The estimated IC50 concentrations of selinexor were approximately 0.1 µM for U266 cells and 0.35 µM for RPMI8226 cells. Moreover, selinexor treatment significantly increased cytotoxicity (Figure 2B), confirming its potent anti-myeloma activity. The observed differences in cytotoxicity between the two myeloma cell lines may reflect inherent biological variation, including genetic background and baseline susceptibility to apoptosis.

### 3.3. AURKA Knockdown Enhancing Selinexor-Induced Cytotoxicity in Myeloma Cells

**Figure 3:**
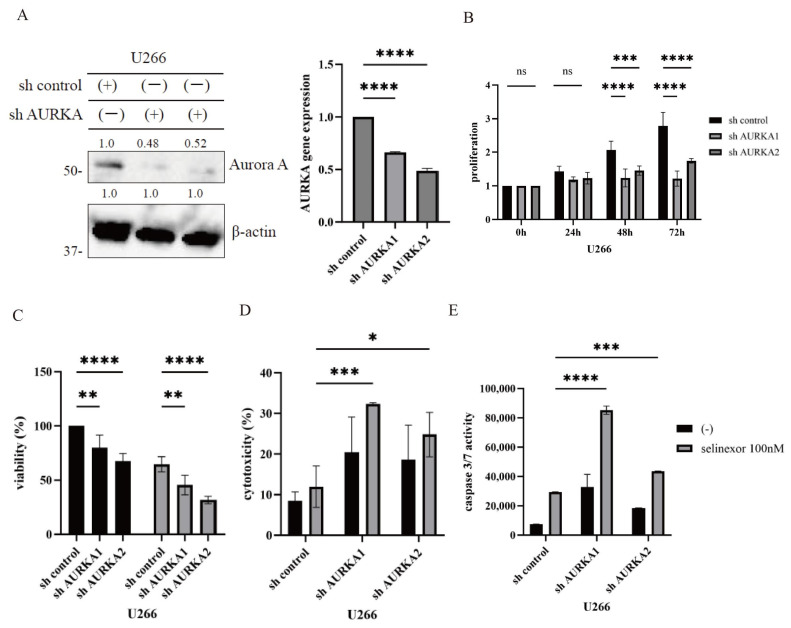
Analysis of the cell proliferation by AURKA shRNA transfection (**A**) AURKA protein expression was assessed by immunoblotting or RT-PCR using a specific anti-AURKA antibody or primer, with β-actin serving as the loading control (*n* = 3). Molecular weight markers are indicated on the left. Band intensities quantified by ImageJ are presented as fold change relative to the control shRNA transfectant cells. (**B**) Cellular proliferation of shRNA-transfected U266 cells was evaluated using trypan blue staining. Significance was indicated as *** < 0.001 and **** *p*  < 0.0001, compared with the control shRNA transfectant cells (*n* = 3). (**C**–**E**) shRNA-transfected U266 cells were treated with 100 nM selinexor for 48 or 72 h. Cell viability (72 h) (**C**), cytotoxicity (48 h) (**D**), and caspase 3/7 activity (48 h) (**E**) were evaluated. * *p*  < 0.05, ** *p*  < 0.01, *** < 0.001 and **** *p*  < 0.0001, compared with the control shRNA transfectant cells (*n* = 3).

shRNA-mediated knockdown was performed in U266 cells to further elucidate the functional role of AURKA. Transfection with shAURKA effectively reduced AURKA expression compared with mock-transfected cells, as demonstrated by immunoblotting and RT-PCR analyses (Figure 3A). AURKA suppression significantly inhibited cellular proliferation in myeloma cells (Figure 3B). Treatment with selinexor further reduced cell viability in both shAURKA-transfected and mock-transfected cells, with shAURKA-transfected cells exhibiting greater sensitivity to selinexor (Figure 3C). Cytotoxicity assays demonstrated a significant increase in apoptosis in shAURKA-transfected cells following selinexor treatment compared with mock controls (Figure 3D). Moreover, caspase-3/7 activity was significantly elevated in shAURKA-transfected cells treated with selinexor (Figure 3E). Collectively, these findings indicate that AURKA suppression enhances the therapeutic efficacy of selinexor in MM cells.

### 3.4. Synergistic Cytotoxic Effects of Combined Selinexor and AURKA Inhibition in Myeloma Cells

**Figure 4:**
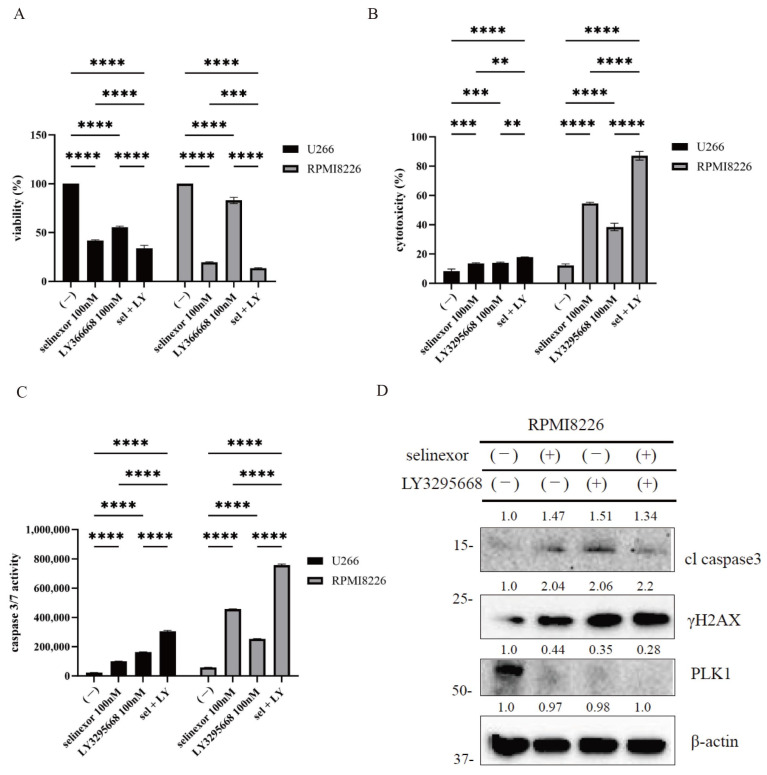
Effects of selinexor alone and in combination with LY3295668 on MM cell lines (**A**–**C**) U266 and RPMI 8226 cells were cultured with selinexor and/or LY3295668 for 48 or 72 h. (**A**) Cell viability (72 h), (**B**) cytotoxicity (48 h), and (**C**) caspase-3/7 activity (48 h) were evaluated. Significance was expressed as ** *p*  < 0.01, *** *p*  < 0.001 and **** *p*  < 0.0001 (*n* = 3). (**D**) RPMI8226 cells were treated with selinexor (100 nM), LY3295668 (100 nM), or their combination for 24 h. Immunoblotting was conducted as described in the Materials and Methods. Blots were probed for cleaved caspase-3, γH2AX, PLK1, and β-actin (loading control) (*n* = 3). Molecular weight markers are indicated on the left. Band intensities quantified by ImageJ are presented as fold change relative to the loading control.

Combined treatment with selinexor and LY3295668 significantly inhibited MM cell growth compared with either agent alone (Figure 4A). Combination index (CI) values calculated using the Chou-Talalay method indicated that selinexor and LY3295668 exerted a synergistic effect (CI < 1). This effect was accompanied by increased cytotoxicity and elevated caspase-3/7 activity, indicative of enhanced apoptotic signaling (Figure 4B,C). Immunoblot analysis showed higher levels of cleaved caspase-3 and γH2AX, together with reduced PLK expression (Figure 4D). Cell cycle analysis showed that LY3295668 caused G_2_/M phase arrest with a concomitant increase in the tetraploid (4N) DNA fraction (Figure S1B). These findings demonstrated that AURKA inhibition—either pharmacologically or genetically—augments the cytotoxic and pro-apoptotic effects of selinexor in MM cells.

### 3.5. Activity of Selinexor and AURK Inhibitor in Bortezomib-Resistant Myeloma and Primary PCL Samples

**Figure 5:**
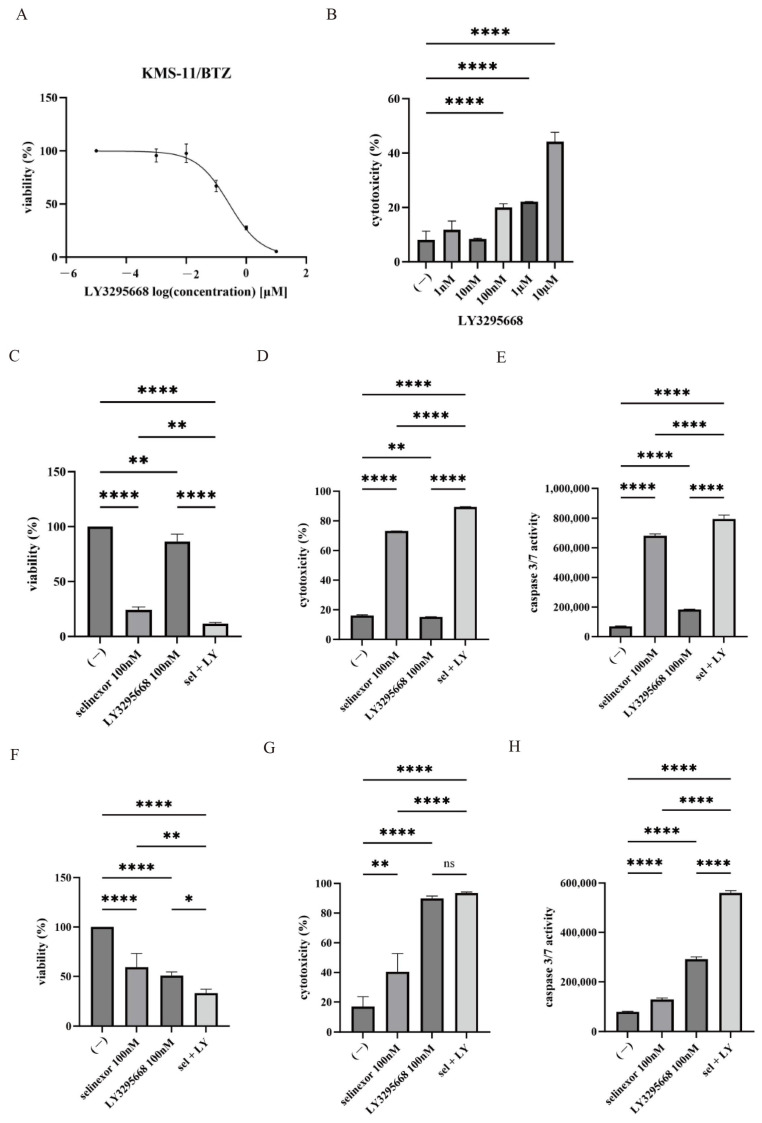
Efficacy of selinexor and/or LY3295668 on the bortezomib-resistant MM cell line and primary PCL samples (**A**,**B**) KMS-11/BTZ cells were cultured with the indicated concentration of LY3295668 for 48 or 72 h. (**A**) Cell viability (72 h) and (**B**) cytotoxicity (48 h) were evaluated. **** *p*  < 0.0001 for the indicated comparisons (*n* = 3). (**C**–**E**) KMS-11/BTZ cells were cultured with selinexor and/or LY3295668 for 48 or 72 h. (**C**) Cell viability (72 h), (**D**) cytotoxicity (48 h), and (**E**) caspase-3/7 activity (48 h) were evaluated. ** *p*  < 0.01 and **** *p*  < 0.0001, for the indicated comparisons (*n* = 3). (**F**–**H**) Primary PCL cells were cultured with selinexor and/or LY3295668 for 48 or 72 h. (**F**) Cell viability (72 h), (**G**) cytotoxicity (48 h), and (**H**) caspase-3/7 activity (48 h) were evaluated. * *p*  < 0.05, ** *p*  < 0.01 and **** *p*  < 0.0001, compared with the untreated sample (*n* = 3).

The effects of selinexor and LY3295668 were further evaluated in the bortezomib-resistant MM cell line KMS-11/BTZ and in primary PCL samples. LY3295668 treatment resulted in a dose-dependent reduction in cell viability in the KMS-11/BTZ cell line, accompanied by a significant increase in cytotoxicity (Figure 5A,B). The estimated IC50 concentration of LY3295668 in KMS-11/BTZ cells was approximately 0.14 µM. Combined treatment with selinexor and LY3295668 further reduced cell viability, enhanced cytotoxicity, and increased caspase-3/7 activity compared with either agent alone (Figure 5C–E). Moreover, the primary PCL samples exhibited sensitivity to LY3295668, and the addition of selinexor did not further heighten cytotoxicity. However, caspase-3/7 activity revealed a modest increase with combination treatment, indicating an enhancement of apoptotic signaling even in the absence of a clear change in cytotoxicity (Figure 5F–H). These findings indicated that the combined use of selinexor and LY3295668 may serve as a promising therapeutic approach for bortezomib-resistant myeloma and aggressive plasma cell disorders, including PCL.

## 4. Discussion

This study shows the therapeutic potential of targeting XPO1 (selinexor) and AURKA (LY3295668) in MM. Both agents induced apoptosis, and their combination produced enhanced cytotoxic and pro-apoptotic effects, underscoring dual inhibition of nuclear export and mitotic regulation as a promising strategy for high-risk MM. Selinexor, already approved for refractory MM and increasingly incorporated into multidrug regimens [23], revealed clear in vitro efficacy in our models, with additional benefit conferred by AURKA inhibition.

AURKA and AURKB are central regulators of cell cycle progression and spindle assembly, and their dysregulation contributes to oncogenesis [16]. Analysis of public GEO datasets showed that AURKA is overexpressed in patient-derived myeloma samples compared with normal controls, with especially elevated expression in plasma cell leukemia, where higher AURKA levels may be linked to worse outcomes. We included these GEO-based findings to corroborate our in vitro data with patient-derived evidence and to underscore the translational relevance of targeting AURKA in MM/PCL. The pharmacological AURKA inhibition by LY3295668 induced caspase-3/7 activation, apoptosis, and cellular senescence, while AURKA knockdown improved MM cell sensitivity to selinexor. Furthermore, selinexor and LY3295668 were effective in primary PCL cells and bortezomib-resistant MM cells, underscoring their activity in clinically challenging disease settings. Mechanistically, selinexor disrupted oncogenic signaling, whereas LY3295668 impaired mitotic progression, collectively promoting enhanced apoptotic responses [24,25].

Several clinical investigations have explored aurora kinase inhibitors in solid and hematologic malignancies. The combination of alisertib and pembrolizumab showed acceptable tolerability and disease stabilization in immunotherapy-resistant patients, whereas AURKA inhibition decreased resistance in retinoblastoma protein-deficient head and neck squamous cell carcinoma [26]. However, combined inhibition of AURKA and the mammalian target of rapamycin yielded limited clinical benefit in patients with refractory solid tumors, including pancreatic adenocarcinoma [27]. Moreover, LY3295668 erbumine exhibited a favorable safety profile and notable antitumor activity in relapsed or refractory neuroblastoma, indicating that biomarker-guided strategies may optimize its clinical application [28]. In breast cancer, the addition of fulvestrant to alisertib failed to improve response rates or progression-free survival; however, alisertib monotherapy showed therapeutic potential in endocrine- and CDK4/6 inhibitor–resistant settings [29]. Collectively, these findings underscore the therapeutic potential of AURK inhibitors, which continue to be explored in both preclinical and clinical studies [30,31,32].

In contrast to previous hematologic studies evaluating bortezomib-based combinations with other AURKA inhibitors, the present approach employed nuclear export blockade to potentiate mitotic stress and DNA damage signaling [33]. Resistance to selinexor may arise through XPO1 overexpression, dysregulation of stress-response pathways, or enhanced drug efflux, whereas resistance to AURK inhibitors can occur via AURKB/PLK1 compensation or TPX2-dependent spindle assembly. Such adaptive mechanisms may attenuate therapeutic efficacy over time, underscoring the importance of careful monitoring [34,35]. Previous work has shown that AURKA inhibition can improve the efficacy of agents such as bortezomib and the BCR::ABL1 inhibitor asciminib, with asciminib plus LY3295668 significantly increasing cytotoxicity in CML cells [36]. In this study, we extend these findings by showing that selinexor-mediated XPO1 inhibition with LY3295668 in MM cells.

This study has some limitations. First, this study is largely based on in vitro experiments and includes only a single PCL patient sample; therefore, the findings should be regarded as exploratory and require validation in relevant in vivo models and clinical settings. Second, most functional assays were conducted in PCL-derived and bortezomib-resistant cells without modeling the bone marrow stromal microenvironment; thus, confirmation in a broader panel of myeloma cell lines and co-culture systems is warranted. Finally, additional studies on toxicity, dose optimization, and the mechanisms underlying the observed synergy will be necessary to refine this approach and for biomarker-guided patient selection.

## 5. Conclusions

Our preclinical data suggest that the combined targeting of nuclear export by selinexor and AURKA by LY3295668 represents a promising therapeutic strategy for MM/PCL and provide a rationale for further clinical investigation of this approach.

## Data Availability

Data supporting the findings of this study are available from the corresponding author upon reasonable request.

## Supplementary Materials

The following supporting information can be downloaded at: https://www.mdpi.com/article/10.3390/hematolrep18010010/s1. Figure S1: Differential AURKA expression in multiple myeloma versus other hematologic cell lines and cell-cycle analysis in multiple myeloma cells.

## Abbreviations

The following abbreviations are used in this manuscript:

AURK	Aurora kinase
AURKA	Aurora kinase A
AURKB	Aurora kinase B
AURKC	Aurora kinase C
BTZ	Bortezomib
CI	Combination index
CRAB	Hypercalcemia, renal impairment, anemia, and bone lesions
DMSO	Dimethyl sulfoxide
FBS	Fetal bovine serum
FDR	False discovery rate
GEO	Gene Expression Omnibus
LOH	Loss of heterozygosity
LDH	Lactate dehydrogenase
MGUS	Monoclonal gammopathy of undetermined significance
MM	Multiple myeloma
PCL	Plasma cell leukemia
PLK	Polo-like kinase
qPCR	Quantitative polymerase chain reaction
RNA	Ribonucleic acid
RT-qPCR	Reverse transcription quantitative polymerase chain reaction
SA-β-gal	Senescence-associated β-galactosidase
shRNA	Short hairpin RNA
TNF-α	Tumor necrosis factor-α
XPO1	Exportin 1
