# Supplementary figures and images for: Therapeutic Potential of Exportin 1 and Aurora Kinase A Inhibition in Multiple Myeloma Cells
Source: Hematol Rep. 2026 Jan 9;18(1):10. doi: 10.3390/hematolrep18010010 (PMC12821534; doi:10.3390/hematolrep18010010)

A

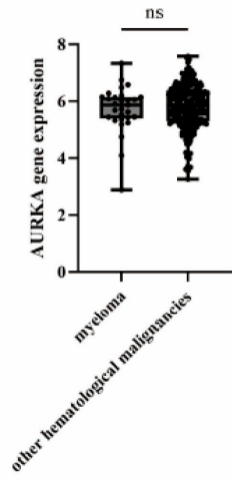

B

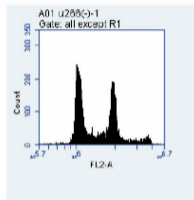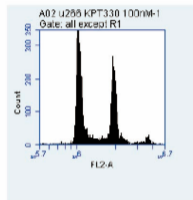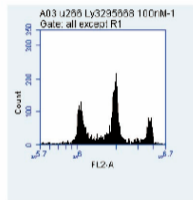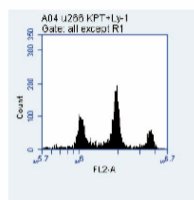

Supplement: Supplementary file 1 [file hematolrep-18-00010-s001.zip › hematolrep-3971584-supplementary.pdf]
